# Sustainable Porous Carbon Materials Derived from Wood-Based Biopolymers for CO_2_ Capture

**DOI:** 10.3390/nano9010103

**Published:** 2019-01-16

**Authors:** Chao Xu, Maria Strømme

**Affiliations:** Division of Nanotechnology and Functional Materials, Department of Engineering Sciences, Uppsala University, SE-75121 Uppsala, Sweden; maria.stromme@angstrom.uu.se

**Keywords:** CO_2_ capture, porous carbon, wood-based biopolymer, cellulose, lignin

## Abstract

Porous carbon materials with tunable porosities and functionalities represent an important class of CO_2_ sorbents. The development of porous carbons from various types of biomass is a sustainable, economic and environmentally friendly strategy. Wood is a biodegradable, renewable, sustainable, naturally abundant and carbon-rich raw material. Given these advantages, the use of wood-based resources for the synthesis of functional porous carbons has attracted great interests. In this mini-review, we present the recent developments regarding sustainable porous carbons derived from wood-based biopolymers (cellulose, hemicelluloses and lignin) and their application in CO_2_ capture.

## 1. Introduction

CO_2_ is the predominant greenhouse gas in the atmosphere. Because of the enormous combustion of fossil fuels during the last two centuries, the atmospheric CO_2_ concentration has dramatically increased from its pre-industrial level of around 280 ppm to today’s 408 ppm (December, 2018). According to a report by the Intergovernmental Panel on Climate Change (IPCC), the excess emission of CO_2_ to the atmosphere is the main reason for the current global climate changes and associated problems, including global warming, sea level rise and ocean acidification, threating human survival and development [1]. Hence, there is an urgent need for reducing the CO_2_ emission and controlling the atmospheric CO_2_ level to mitigate climate changes. 

It is believed that fossil fuels will still dominate the global energy system in the foreseeable future. In this context, carbon capture and storage (CCS) is considered as a feasible and necessary approach to reduce CO_2_ emission [2]. CCS covers a group of technologies including capture, compression, transportation and permanent storage of CO_2_ [3]. Herein, CO_2_ capture is the initial and the most important step. Conventional CO_2_ capture processes address large CO_2_ emission sources like flue gas emitted from coal-fired power plants, cement plants, oil refinery factories and iron and steel plants. Of the several technologies being developed for conventional CO_2_ capture processes, post-combustion capture of CO_2_ is a straightforward and commercially mature CO_2_ capturing technique that can be easily retrofitted to existing power plants. It separates CO_2_ from the flue gases, which consists of about 5–15 v% CO_2_, 70–75 v% N_2_, 5–7 v% H_2_O, 3–4 v% O_2_, and NO_x_ and SO_x_ with low concentrations (a few hundred ppm) [4]. The low pressure of the flue gases (ca. 1 atm) and their low CO_2_ concentration require a high separation efficiency for CO_2_ over other gas components at ambient pressure. Amine scrubbing, an absorption technique using aqueous alkanolamines solution to remove CO_2_ from gas streams, has been applied industrially in natural gas purification and post-combustion capture of CO_2_ for more than 50 years [5]. The chemical reaction of CO_2_ with amine forms stable carbamates, offering a high efficiency for CO_2_ capture and separation. However, it suffers from several drawbacks of high energy penalties for the amine regeneration, risk of amine leakage and corrosion to the employed equipment. 

**Scheme 1:**
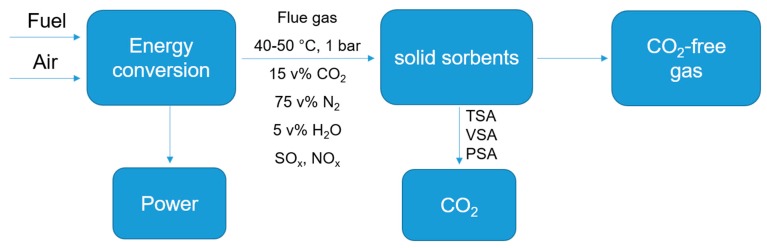
Post-combustion capture of CO_2_ from flue gas using solid sorbents. TSA = temperature swing adsorption; VSA = vacuum swing adsorption; PSA = pressure swing adsorption.

Recently, large efforts have been devoted to the development of adsorption-driven separation techniques using porous materials as solid sorbents for CO_2_ capture, which is considered an alternative approach to the amine scrubbing process [6,7,8]. Porous materials are a type of solid containing pores, usually interconnected, or channels possessing a high surface area [9]. Typical examples are traditional zeolites, activated carbons, mesoporous oxides and emerging metal-organic frameworks (MOFs), covalent-organic frameworks, porous organic polymers, etc. Different from the chemical absorption approach, a typical adsorption process starts by attracting CO_2_ molecules onto the large surface of the porous solid by physisorption and/or chemisorption interactions. To regenerate the solid adsorbent and remove and concentrate the CO_2_, the CO_2_-filled solid is exposed to temperature, vacuum or pressure swing adsorption (TSA, VSA, PSA) cycles (Scheme 1) [10]. Because of the much weaker CO_2_-adsorbent interactions taking place in the above described process than in CO_2_-amine interactions/chemical reactions, the adsorption-driven separation technique requires significantly lower amounts of energy in the regeneration procedure compared with the amine-scrubbing process. 

In general, CO_2_ adsorption capacity mainly depends on intrinsic characteristics (e.g., surface area, pore size and adsorption sites) of the porous material, while the adsorption selectivity is influenced by molecular sieving, thermodynamic equilibrium and kinetic effect. Therefore, all of these factors have to be taken into account for the design and selection of porous materials as CO_2_ adsorbents towards a high CO_2_ adsorption capacity and a high adsorption selectivity of CO_2_-over-other gases (e.g., N_2_, H_2_O, CH_4_). Other factors such as cost, stability and processability of the material should also be considered when it comes to practical applications. For example, zeolites, crystalline microporous aluminosilicates, are widely used in industrial CO_2_ capture. They usually display high CO_2_ adsorption capacities and high CO_2_-over-N_2_ selectivities because of their uniform and narrow pore sizes [11]. However, most zeolites are hydrophilic and drastically lose their adsorption capacity and selectivity for CO_2_ under humid conditions. Therefore, the flue gas needs to be dried prior to passing through the zeolite sorbents for separating CO_2_, which significantly increase the operation cost. MOF materials are coordination polymers joining organic linkers and metal ions or clusters that have been intensively studied for CO_2_ adsorption and separation. The use of reticular chemistry principles allows for the design of MOF materials at the molecular level with tailored crystal structure, pore size and functional groups for the development of highly efficient CO_2_ sorbents [12,13]. However, MOF materials in this context suffer from low stability and high manufacturing cost. In comparison, porous carbons (or activated carbons) with high surface areas, tunable pore sizes and high tolerance to acidic, basic and humid environments are considered to be ideal sorbents for CO_2_ capture [14,15]. More significantly, porous carbons can be prepared from sustainable biomass precursors providing an environmentally friendly and sustainable approach for the development of CO_2_ sorbents [16]. 

Wood, the largest biomass resource on earth, has been used for tools, fuels and buildings throughout human history. Apart from these traditional functions, wood has been studied as a basis for functional materials during the development of modern material science and nanotechnology in recent years [17,18]. Cellulose, hemicellulose and lignin are biopolymers that constitute the main components of wood and many other plants. From a chemistry point of view, cellulose is a linear polysaccharide consisting of repeated D-glucose unit with the formula of (C_6_H_10_O_5_)_n_. Hemicellulose is a branched polysaccharide containing different sugar monomers. In comparison, lignin has a more complex composition and lacks a defined structure. It is basically an aromatic polymer network of cross-linked phenylpropane derived lignols. Because of their rich carbon content, natural abundance, and low cost, all these three biopolymers have been used as precursors to prepared porous carbons. This paper will review recent studies on sustainable porous carbon materials derived from the wood-based biopolymers cellulose, hemicellulose and lignin and their applications in CO_2_ capture. The conversion route of biopolymers to porous carbons and the related mechanisms are introduced. The relationship between CO_2_ adsorption behaviors (e.g., CO_2_ adsorption capacity, CO_2_-over-N_2_ selectivity, kinetics) and nanostructures (porosity, composition) of the biopolymer-based porous carbons are summarized. Additionally, we present our perspectives on the challenges and future developments of using sustainable porous carbon materials in CCS. 

## 2. General Routes from Biomass to Porous Carbons

The conversion of biomass to porous carbons usually consists of two processes: carbonization and activation [19]. A conventional carbonization process is usually carried out in an inert atmosphere (e.g., N_2_, Ar) at elevated temperatures of 400–1000 °C, which is a typical pyrolytic approach. It involves several complex reactions such as dehydration, condensation and isomerization, by which most of the hydrogen and oxygen atoms are released from the biomass to form H_2_O, H_2_, CH_4_, CO gases and various violates while the carbon atoms are condensed as solid residues (usually denoted as char) with an increased carbon content [20]. The yield and carbon content of the solid are affected by several factors such as carbonization temperature, resistance time, heating rate, chemical structure and thermal stability of the biomass. In general, an increased carbonization temperature and long resistance time form chars with a reduced yield but elevated carbon content [21]. Apart from the conventional pyrolysis process, hydrothermal carbonization (HTC) is another approach that has been widely applied in the conversion of biomass to valuable carbon materials. The treatment of biomass under HTC conditions, usually at moderate temperatures (<300 °C) and self-generated pressures, forms hydrochars with increased C/H and C/O ratios and can be considered as an accelerated coalification process [22,23,24,25]. In contrast to the traditional pyrolytic carbonization method, the HTC process has several advantages. First, it operates under moderate temperatures, which significantly reduces the energy consumption. Second, wet biomass precursors do not need drying and can be directly subjected to the HTC treatment because the hydrothermal process takes place in water. In addition, the conversion of biomass by the HTC process gives carbon-rich hydrochars in high yields. Furthermore, the carbonaceous materials can be complexed with other components such as noble metal nanoparticles, magnetic nanoparticles, and electrochemically active species during the HTC process to form functional nanocomposites with desired nanostructures and compositions displaying a variety of physiochemical properties and functionalities. Therefore, the HTC method not only offers an energy-saving and environmentally friendly approach for biomass carbonization, but also significantly contributes to the development of carbon-based functional nanomaterials. 

The chars obtained from the carbonization process are usually nonporous. Therefore, further activation, either physically or chemically, of the chars at elevated temperatures is a necessary step for the preparation of highly porous carbon materials. The physical activation method uses oxidizing atmosphere such as CO_2_, air or steam, while chemical activation applies activating agents such as KOH, NaOH, KHCO_3_, K_2_CO_3_, ZnCl_2_ or H_3_PO_4_ [26,27,28]. Certain moieties of the chars are prone to be oxidized and dehydrated by the oxidizing gases or chemical reagents during the activation process, and thus, create rich micro- and mesopores [21]. The two activation methods produce porous carbons with large differences in porosity. In general, physical activation processes create porous carbons with moderate surface areas (<1000 m^2^/g) and narrow micropores that can be beneficial for, e.g., CO_2_/N_2_ and CO_2_/CH_4_ separation [29]. In contrast, chemical activation methods can significantly increase the surface area (up to >3000 m^2^/g) and pore volume of the porous carbons that may be useful for applications in gas storage [30,31], water treatment [32,33], electrochemical supercapacitors [34,35], etc. In addition, chemical activation methods allow for conversion of biomass to highly porous carbons in a one-step carbonization/activation process, which greatly facilitates the manufacturing process without involving several procedures [36]. However, most chemical activation methods suffer from significant environmental disadvantages related to their dependence on large amounts of corrosive activating agents [37]. 

## 3. Synthesis of Porous Carbons from Wood-Based Biopolymers

**Figure 1:**
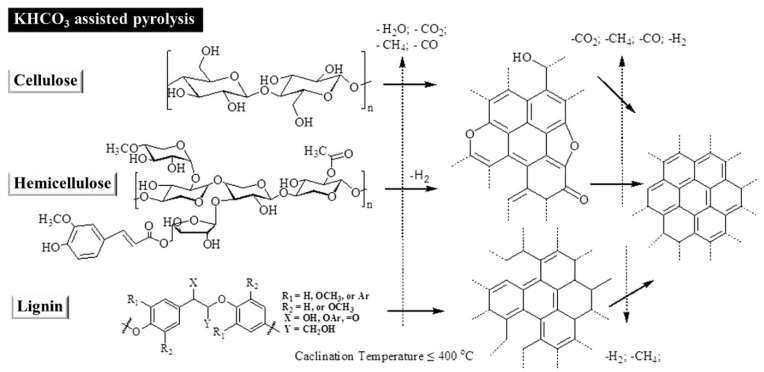
Possible carbonization routes of cellulose, hemicellulose and lignin in the presence of KHCO_3_ (reproduced from [40], with permission from American Chemical Society, 2016).

Both traditional pyrolysis and hydrothermal treatment have been applied in the carbonization of wood-based biopolymers. The carbonization process and the obtained carbonaceous solid have been well studied with the assistance of various analytical techniques. For example, the combination of thermogravimetry (TG) and gas chromatography (GC)/mass spectrometry (MS) is useful to probe the pyrolysis behavior and analyze the gaseous products. Yang et al. have conducted TG analyses for cellulose, hemicellulose and lignin under N_2_ atmosphere [38]. These studies indicated that hemicellulose, having the lowest thermal stability among the three biopolymers because of its random amorphous structure and low molecular weight, starts to decompose at 220 °C. Cellulose decomposes between 315 and 390 °C. The relatively high thermal stability of cellulose can be attributed to its crystalline structures, strong hydrogen boding between cellulose chains, and high molecular weight. In contrast, lignin has a wide and flat TG curve in the range of 250–900 °C. The slow pyrolysis rate and high thermal stability of lignin can be explained by its heavily cross-linked and aromatic-rich network. The solid yield of hemicellulose, cellulose and lignin at a temperature of 900 °C was ~20%, 7% and 40%, respectively. The overall low solid yields can be attributed to the preferred gasification of the biopolymers at high temperatures with formation of rich gaseous products. Kwon et al. have studied the effects of gas atmosphere and heating rate on the pyrolytic carbonization of cellulose [39]. They showed that the use of different atmosphere (N_2_ and CO_2_) does not influence the solid yield at the same heating rate. However, the use of CO_2_ for the carbonization significantly enhanced the generation of gaseous products including H_2_, CO and CH_4_ because CO_2_ could promote the thermal cracking behaviors, which indicates that the use of CO_2_ as a reaction medium gave high thermal efficiency for biomass conversion. On the other hand, an increase of the heating rate from 10 °C/min to 500 °C/min reduced the solid yield from 17% to 8% at 900 °C, which is consistent with the findings from other studies that a high heating rate usually leads to a low solid yield for biomass conversion. Deng et al. applied TG-MS tools to investigate the process of carbonization/activation of cellulose, hemicellulose and lignin in the presence of activating agent of KHCO_3_ [40]. The TG analysis showed that KHCO_3_ decomposed at 200°C and KHCO_3_ accelerated the pyrolysis of biopolymers. At temperatures above 400 °C, the byproducts of KHCO_3_ (e.g., K, K_2_CO_3_, and K_2_O) catalyzed the activation process with the formation of H_2_ and CH_4_ as detected by MS. Based on these observations, this study proposed a possible structure evolution of the biopolymers during the carbonization/activation process, as shown in Figure 1. Interestingly, many studies have shown that cellulose can be transformed to porous carbons in a single step of carbonization under inert gas (N_2_ or Ar) without the use of any oxidizing gases or activating agents. Bommier et al. synthesized a series of porous carbons with high surface areas by carbonization of filter paper under Ar atmosphere [41]. Based on TG-MS studies that monitored the carbonization process, they proposed a self-activation mechanism: the obtained carbon material was in-situ activated by the gaseous products (e.g., H_2_O, CO_2_) from the decomposition of cellulose. The in-depth investigation of the thermal degradation process presented in these studies allows us to understand the conversion mechanisms and to optimize the carbonization parameters, such as temperature, heating rate, resistance time and atmosphere for the synthesis of carbon materials with desired properties. 

**Figure 2:**
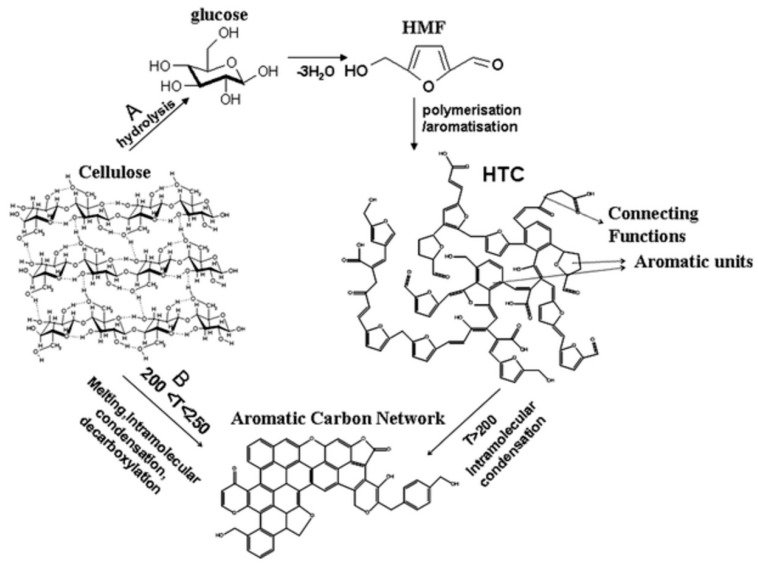
Conversion of cellulose to aromatic carbons under hydrothermal carbonization (HTC) conditions (reproduced from [22], with permission from The Royal Society of Chemistry, 2012).

As mentioned above, the HTC process has the advantage of operating at moderate temperatures, forming hydrochars with a rich variety of organic functional groups, allowing for the study of their molecular details by various spectroscopic and electron microscopic tools in order to investigate the details of the carbonization mechanism. Falco et al. have investigated the effects of processing temperature and time on morphology and chemical structure of the HTC hydrochars generated from cellulose [42]. X-ray diffraction, solid-state ^13^C nuclear magnetic resonance (NMR) spectroscopy and scanning electron microscopy studies of the hydrochars revealed that the fibrous and crystalline structure of the cellulose was unaffected by hydrothermal treatment at low operating temperatures (<160 °C). Upon increasing the temperature, the fibrous structure started to decompose with formation of spherical particles. When the cellulose was treated at higher temperatures (200–280 °C), *sp*^2^, hybridized carbons with moieties such as O−C=O, C=C−C were identified in the solid-state ^13^C-NMR spectra of the obtained hydrochars. Based on these observations, two possible routes were proposed for the conversion of cellulose to hydrochars under HTC conditions (Figure 2). One route involved (a) hydrolysis of cellulose into glucose, (b) dehydration of glucose into hydroxymethylfurfural (HMF), (c) polymerization and polycondensation of HMF into polyfuranic chains, (d) intramolecular condensation, dehydration and decarboxylation of polyfuranic chains into aromatic rich carbon network via reactions. The other route was the direct aromatization of cellulose that forms aromatic carbon networks at higher HTC processing temperatures (200–280 °C); similar to a classical pyrolysis process. Other mechanistic routes have also been proposed for HTC conversion of various biopolymers by means of infrared, Raman and X-ray photoelectron spectroscopy [43,44,45,46]. These studies have greatly promoted the understanding of chemical reactions occurring during the HTC process, which enables tailoring of the nanostructure, porosity and functionality of carbon materials.

## 4. CO_2_ Adsorption on Cellulose-, Hemicellulose- and Lignin-Derived Porous Carbons

As mentioned, cellulose can be converted to porous carbon material in a one-step pyrolytic carbonization process under N_2_ or Ar atmosphere and physical or chemical activation of the carbonized solid could further increase its surface area and enhance its CO_2_ adsorption capacity. For example, Heo et al. reported a series of porous carbons derived from commercial cellulose fibers in three steps: pre-pyrolysis under N_2_ atmosphere at 200 °C, carbonization under N_2_ atmosphere at 750–800 °C and physical activation with steam [47]. Morphology studies indicated that steam molecules played a key role in the pore-opening process and induced an increase in the surface area of the porous carbons formed. Meanwhile, the physical activation process significantly contributed to the evolution of ultramicropores (pore size < 0.8 nm). As a result, the surface areas were increased from 452–540 m^2^/g for pre-activated samples to 599–1018 m^2^/g for steam-activated samples. Accordingly, steam activation led to increase in CO_2_ adsorption capacity and CO_2_-over-N_2_ selectivities. Recently, we have applied *Cladophora* cellulose, a type of nanofibrous cellulose extracted from algae, and its chemically modified derivatives as precursors to prepare highly porous carbons [48]. The cellulose precursors were treated in a one-step carbonization/activation approach at 900 °C under N_2_ or CO_2_ atmosphere. The porous carbons activated in CO_2_ had significantly higher surface areas (832–1241 m^2^/g) and higher volumes of ultramicropores (0.24–0.29 cm^3^/g) than those prepared in N_2_ (393–500 m^2^/g; 0.11 cm^3^/g), due to the fact that CO_2_ could activate the precursors to a higher extent than N_2_ could, and hence, induce the formation of more (ultra-)micropores. As a result, the CO_2_ adsorption capacity of the porous carbons was remarkably increased by the CO_2_ activation approach. In another related study, Zhuo et al. prepared hierarchically porous carbons by carbonization/activation of cellulose aerogels under CO_2_ and N_2_ atmosphere, respectively [49]. The CO_2_-activated porous carbon had significantly higher surface area, higher volume of micropores and higher CO_2_ adsorption uptake than the N_2_-carbonized porous carbon. These results suggest that CO_2_ or steam activation is an efficient approach to prepared cellulose-based porous carbons with high CO_2_ adsorption capacities. 

In comparison with physically activated carbons, chemically activated carbons have much higher surface areas, and thus, generally show relatively higher CO_2_ adsorption capacities. For example, Sevilla et al. reported on chemical activation of hydrothermally carbonized cellulose by KOH [16]. The obtained microporous carbon had a very high surface area of 2370 m^2^/g and a relatively high CO_2_ adsorption capacity of 5.8 mmol/g at 1 bar and 273 K. In addition, the carbon exhibited a high CO_2_ adsorption rate and excellent adsorption recyclability. Wang et al. used hemp stem hemicellulose as precursor and prepared well-shaped porous carbon spheres by a HTC process and a subsequent KOH activation procedure. The obtained porous carbon spheres had large surface areas of up to 3062 m^2^/g and high CO_2_ adsorption capacities of up to 5.63 mmol/g (1 bar, 273 K) [50]. Hao et al. reported on the treatment of lignin under HTC conditions at 360–385 °C. Further activation of the hydrocarbon by KOH gave highly porous carbons with surface areas of up to 2875 m^2^/g and high CO_2_ adsorption capacities of up to 6.0 mmol/g (1 bar, 273 K) [51]. There are many activation parameters that influence the porosity and CO_2_ adsorption behaviors of wood-biopolymer derived porous carbons. For example, Sangchoom et al. showed that activation at a high KOH/carbon ratio led to increase in the surface area and total pore volume for lignin-derived porous carbons; however, the CO_2_ adsorption capacities and the ultramicropore volume decreased with increasing KOH/carbon ratio [52]. Balahmar et al. prepared lignin-based porous carbons by a novel mechanochemical activation method based on compaction of lignin precursors and KOH at a high pressure of 740 MPa prior to thermal activation. The compact contact between the lignin precursor and the activating agent resulted in enhanced surface area, pore volume and CO_2_ adsorption capacity [53]. It should be noted that chemically activated carbons usually have broad pore size distributions and a high degree of mesporosity. Therefore, their CO_2_-over-N_2_ selectivities are largely sacrificed by the high surface areas. 

Apart from surface area and pore volume, N-doping plays an important role in CO_2_ adsorption for porous carbon materials. In order to investigate the effect of N-doping on CO_2_ adsorption behaviors for cellulose-based porous carbons, Hu et al. prepared N-doped and N-free carbon aerogels by activation/carbonization of cellulose aerogels at high temperatures (700–900 °C) under NH_3_ or N_2_ atmosphere, respectively [54]. Notably, the N-doped carbon aerogel showed 40% higher CO_2_ adsorption capacity than that of the N-free carbon aerogel (4.99 versus 3.56 mmol/g at 1 bar, 298 K). In addition, Demir et al. and Saha et al. prepared N-doped porous carbons from lignin. All these N-doped porous carbons showed relatively high CO_2_ adsorption capacities up to 8.6 mmol/g (1 bar, 273 K) [55,56]. The high CO_2_ adsorption capacity of the N-doped carbon materials can be explained by various interactions (e.g., Lewis acid-Lewis base interaction, hydrogen bonding interaction) between the N-containing species and the CO_2_ molecules. 

Besides using pristine biopolymers, there are some examples in the literature employing chemically modified biopolymers as precursors for the preparation of porous carbons. In a recent study, we reported chemical modification of *Cladophora* cellulose [48]. The pristine cellulose was first oxidized to dialdehyde cellulose (DAC) by using sodium metaperiodate as the oxidizing agent. The DAC was further reacted with chitosan, a biopolymer containing amine groups, to form a cross-linked cellulose (CLC) via polycondensation reactions. As a result, a series of porous carbons with tunable porosity were synthesized. Interestingly, the cross-linked structure in the cellulose precursor resulted in slightly decreased surface area, however, it created a high degree of ultramicropores in the obtained porous carbons. Hence, the CO_2_ adsorption capacity (3.39 mmol/g for CLC-carbon versus 2.64 mmol/g for cellulose-carbon; 1 bar, 273K) and CO_2_-over-N_2_ selectivity (42 for CLC-carbon versus 32 for cellulose-carbon; 273 K) were significantly increased. In addition, lignin can be also chemically modified to further increase the cross-linking. For example, Meng et al. reported hypercrosslinking of organosolv lignin with formaldehyde dimethyl acetal as crosslinker in a Friedel–Crafts reaction [57]. The obtained lignin had moderate CO_2_ adsorption capacity but excellent CO_2_-over-N_2_ selectivity. Pyrolysis of the hypercrosslinked lignin created microporous carbons with increased CO_2_ capacity and relatively high selectivity. These studies demonstrate that introducing cross-linking structures into biopolymers can facilitate the formation of micropores in the derived carbon materials. Therefore, chemical modification of biopolymers allows us to tailor the precursor structures at the molecular level, and thus, to optimize the porosity and enhance the CO_2_ capture efficiency for the corresponding porous carbon materials.

**Figure 3:**
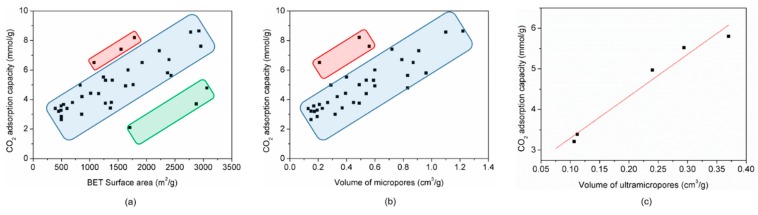
Plots of (**a**) CO_2_ uptake (1 bar, 273 K) versus BET surface area; (**b**) CO_2_ uptake (1 bar, 273 K) versus volume of micropores; (**c**) CO_2_ uptake (1 bar, 273 K) versus volumes of ultramicropores for cellulose-, hemicellulose- and lignin-derived porous carbons.

Table 1 lists literature reported cellulose-, hemicellulose- and lignin-derived porous carbons. Their activation method, surface areas, volume of (ultra-)micropores, CO_2_ adsorption capacities at different partial pressures, CO_2_-over-N_2_ selectivity and heat of adsorption (*Q*_st_) of CO_2_ are summarized. For comparison, other selected sorbents of porous polymer, MOF and zeolite are also included. Based on these data, we have correlated the CO_2_ adsorption capacity with surface area and pore volume of these biopolymer-derived porous carbons. Figure panels 3a and b show CO_2_ uptake (1 bar, 273 K) versus Brunauer–Emmett–Teller (BET) surface area and CO_2_ uptake (1 bar, 273 K) versus volume of micropores, respectively. Obviously, most of the data points in the two panels are confined within the blue narrow rectangular areas, indicating that there is a significant correlation between CO_2_ uptake and surface area as well as between CO_2_ uptake and micropore volume. However, the correlations are not strictly linear: for example, the data points in the green rectangle (Figure 3a) indicate that the corresponding porous carbons (MAC-S-8^51^, MAC-E-8^51^, HACS-5^50^.) have high surface areas but relatively low CO_2_ uptakes. This can be attributed to the lack of ultramicropores in the porous carbons. In contrast, the data points in the red rectangles (Figure 3a,b) suggest that the corresponding carbon materials (LHPC-700^55^, LHPC-850^55^, LHPC-1000^55^, LAC2700^52^) have relatively high CO_2_ adsorption capacities, although their surface areas and volumes of micropores are moderate. The relatively high CO_2_ adsorption capacities can be attributed to the N-doped structures and the large volumes of ultramicropores. In addition, the plot of CO_2_ uptake (1 bar, 273 K) versus volumes of ultramicropores in Figure 3c clearly demonstrates that the CO_2_ uptake at 1 bar correlates strongly with the volume of ultramicropores, which is consistent with previous studies [58,59]. These results suggest that formation of large amounts of ultramicropores and N-doping structure in the porous carbons are efficient approaches to reach a high CO_2_ adsorption capacity. Based on these reported data, it is estimated that 1 ton of biopolymer-based porous carbon could capture up to 50 kg of CO_2_ at real conditions for postcombustion capture of CO_2_ (0.15 bar, 40 °C) from flue gas. Apart from CO_2_ adsorption capacity, CO_2_-over-N_2_ selectivity and heat of adsorption (*Q*_st_) of CO_2_ are both important for industrial CO_2_ capture. Notably, the physically activated carbons have much higher CO_2_-over-N_2_ selectivities (26-47) than those of the chemically activated carbons (5.4–25), because the former contain narrow (ultra-)micropores while the latter usually have broad pore size distributions and possess large amounts of meso- and macropores, and thus, reduce the molecular sieving effects. All these porous carbons have moderate *Q*_st_ (CO_2_) values of 20–41 kJ/mol, revealing that physical interactions govern the adsorption of the CO_2_ molecules on the carbon materials. Compared to chemical adsorption, physical adsorption significantly speeds up the adsorption kinetics and facilitates regeneration of the sorbents under swing adsorption conditions [60].

## 5. Conclusions and Perspectives

This review highlights the utilization of the wood-based biopolymers cellulose, hemicellulose and lignin as precursors to prepare sustainable and efficient porous carbons as CO_2_ sorbents. Such porous carbons can be manufactured at large scale in low cost for practical industrial applications thanks to the abundance and low price of these biopolymers. The advantages of using such sustainable porous carbons for industrial CO_2_ capture include high CO_2_ adsorption capacity, high physiochemical stability, easy regeneration, fast adsorption/desorption rate, low operation cost and low manufacturing cost. However, most of these porous carbons have CO_2_-over-N_2_ selectivities that are lower than those of other CO_2_ sorbents, such as zeolite, MOFs and porous polymers. This limitation can be overcome by tailoring the nanostructures of the porous carbons, for example, by introducing hetero-atoms and CO_2_-philic species, formation of large amounts of narrow ultramicropores promoting the CO_2_ adsorption kinetics and/or thermodynamics. Intensive studies have been devoted to tailoring the porosity of porous carbons by controlling the carbonization/activation conditions. However, the relationship between the molecular structure of the biopolymers and the properties of the porous carbons is still unrevealed. Future research could focus on such structure-property relationships in order to gain control over the properties of biopolymer-based porous carbons at the molecular level towards development of highly efficient CO_2_ sorbents. 

## Figures and Tables

**Table 1:** A summary of biopolymer-based porous carbons and their textural properties, CO_2_ uptake, CO_2_/N_2_ selectivity and heat of adsorption. Other selected sorbents (porous polymer, metal-organic frameworks (MOF), zeolite) are also included for comparison.

Sample	Biopolymer	Activating Agent	S_BET_ (m^2^/g)	V_micropores_ (cm^3^/g) *^a^*	CO_2_ Uptake (mmol/g) *^b^*		S *^c^*	*Q*_st_ (kJ/mol)	Ref.
					1 bar	0.15 bar			
**CA800**	**Cellulose**	**N_2_**	**496**	**0.17**	**3.56**	-	-	-	[54]
CF-700	Cellulose	N_2_	499	0.193	2.846	-	28.7	27.2	[47]
CF-750	Cellulose	N_2_	494	0.193	3.271	-	26.4	24.5	[47]
CF-800	Cellulose	N_2_	540	0.209	3.664	-	27.6	25.1	[47]
CF-850	Cellulose	N_2_	452	0.174	3.189	-	27.9	25.7	[47]
CF-700-act	Cellulose	CO_2_	599	0.229	3.395	-	35.1	29.5	[47]
CF-750-act	Cellulose	CO_2_	696	0.267	3.792	-	36.7	31.0	[47]
CF-800-act	Cellulose	CO_2_	863	0.334	4.192	-	47.1	37.8	[47]
CF-850-act	Cellulose	CO_2_	1018	0.393	4.416	-	39.2	33.4	[47]
LCN-1	Lignin	KOH	2922	1.22	8.64	3.2	-	40.0	[56]
LCN-2	Lignin	KOH	2779	1.10	8.56	3.03	-	32.5	[56]
LCN-3	Lignin	KOH	1631	0.60	4.92	1.88	-	41.0	[56]
L2600	Lignin	KOH	1277	0.59	5.3	1.3	-	-	[53]
L2600P	Lignin	KOH	2224	0.91	7.3	2.3	-	-	[53]
MAC-E-7	Lignin	KOH	1674	0.60	6.0	1.8	15.0	30.0	[51]
MAC-E-8	Lignin	KOH	2875	-	3.7	0.9	16.0	-	[51]
MAC-S-7	Lignin	KOH	1380	0.45	3.8	1.1	11.0	-	[51]
MAC-S-8	Lignin	KOH	1706	-	2.1	0.5	11.0	-	[51]
HACS-1	Hemicellulose	KOH	1276	0.49	3.75	0.68	-	-	[50]
HACS-2	Hemicellulose	KOH	1397	0.54	5.31	1.44	-	-	[50]
HACS-3	Hemicellulose	KOH	1764	0.49	5.00	1.37	-	-	[50]
HACS-4	Hemicellulose	KOH	2431	0.83	5.63	1.16	-	-	[50]
HACS-5	Hemicellulose	KOH	3062	0.83	4.78	0.85	-	-	[50]
LHPC-700	Lignin	KOH	1788	0.49	8.2	2.48	21.8	28.6	[55]
LHPC-850	Lignin	KOH	2957	0.56	7.6	2.26	15.6	28.4	[55]
LHPC-1000	Lignin	KOH	1075	0.21	6.5	2.07	13.5	27.3	[55]
PPC-850	Lignin	KOH	2396	0.79	6.7	1.97	10.8	27.6	[55]
Cell-N_2_	Cellulose	N_2_	859	0.32	3.00	0.82	-	-	[49]
Cell-CO_2_	Cellulose	CO_2_	1364	0.37	3.42	1.02	-	-	[49]
AC-4-700	Cellulose	KOH	2370	0.96 (0.37)	5.80	1.48	5.4	20.0	[16]
LAC2600	Lignin	KOH	1157	0.54	4.4	-	24.5	-	[52]
LAC2700	Lignin	KOH	1551	0.72	7.4	-	25.0	-	[52]
LAC2800	Lignin	KOH	1924	0.87	6.5	-	20.5	-	[52]
CC-AC-N_2_	cellulose	N_2_	500	0.15	2.64	1.30	32.6	31.5	[48]
DAC-AC-N_2_	Dialdehyde cellulose	N_2_	455	0.15 (0.11)	3.21	1.66	40.5	26.8	[48]
CLC-AC-N_2_	Cross-linked cellulose	N_2_	393	0.13 (0.11)	3.39	1.82	41.8	29.9	[48]
DAC-AC-CO_2_	Dialdehyde cellulose	CO_2_	1241	0.40 (0.29)	5.52	1.96	28.4	32.1	[48]
CLC-AC-CO_2_	Cross-linked cellulose	CO_2_	832	0.29 (0.24)	4.97	2.29	32.9	28.5	[48]
**MOP A-B1**			378	0.11	2.68	1.20	68	29.0	[61]
**Cu-TDPAT**			1938	0.93	10.1	2.60	300	42.2	[62]
**Zeolite 13 *^d^***			616	0.34	4.80	3.50	-	-	[63]

*^a^* The values in the parentheses are volume of ultramicropores calculated from the N_2_ adsorption isotherms; *^b^* data collected at 273 K; *^c^* Selectivity of CO_2_-over-N_2_; *^d^* CO_2_ adsorption data was recorded at 288 K. (-): data not available.
